# Viruses in astrobiology

**DOI:** 10.3389/fmicb.2022.1032918

**Published:** 2022-10-26

**Authors:** Ignacio de la Higuera, Ester Lázaro

**Affiliations:** ^1^Department of Biology, Center for Life in Extreme Environments, Portland State University, Portland, OR, United States; ^2^Centro de Astrobiología (CAB), CSIC-INTA, Torrejón de Ardoz, Spain

**Keywords:** astrobiology, virosphere, origin of life, quasispecies, horizontal gene transfer, virus biosignatures, experimental evolution

## Abstract

Viruses are the most abundant biological entities on Earth, and yet, they have not received enough consideration in astrobiology. Viruses are also extraordinarily diverse, which is evident in the types of relationships they establish with their host, their strategies to store and replicate their genetic information and the enormous diversity of genes they contain. A viral population, especially if it corresponds to a virus with an RNA genome, can contain an array of sequence variants that greatly exceeds what is present in most cell populations. The fact that viruses always need cellular resources to multiply means that they establish very close interactions with cells. Although in the short term these relationships may appear to be negative for life, it is evident that they can be beneficial in the long term. Viruses are one of the most powerful selective pressures that exist, accelerating the evolution of defense mechanisms in the cellular world. They can also exchange genetic material with the host during the infection process, providing organisms with capacities that favor the colonization of new ecological niches or confer an advantage over competitors, just to cite a few examples. In addition, viruses have a relevant participation in the biogeochemical cycles of our planet, contributing to the recycling of the matter necessary for the maintenance of life. Therefore, although viruses have traditionally been excluded from the tree of life, the structure of this tree is largely the result of the interactions that have been established throughout the intertwined history of the cellular and the viral worlds. We do not know how other possible biospheres outside our planet could be, but it is clear that viruses play an essential role in the terrestrial one. Therefore, they must be taken into account both to improve our understanding of life that we know, and to understand other possible lives that might exist in the cosmos.

## Introduction

Astrobiology is a scientific discipline concerned with the origin, evolution, distribution and future of life in the universe (Des Marais et al., 2008; Hays et al., 2015; Cockell, 2020; O’rourke et al., 2020; Lingam and Loeb, 2021; Bennett et al., 2022). The use of the term life in reference to the object of study of astrobiology may raise doubts about whether viruses should be included in its field of research. Some of the difficulties in deciding this question derive from the absence of a universal definition of life (Dix, 2003; Benner, 2010; Cleland, 2011, 2019), which is partly due to the fact that the only example of life we know is that on Earth. That all living beings on this planet share the same ancestor (Woese et al., 1990) makes it difficult to identify the fundamental properties of life. What we think is essential because of its presence in all forms of known life, may simply represent a characteristic that has been inherited from the common progenitor.

NASA defines life as a self-sustaining chemical system capable of darwinian evolution. Although viruses are chemical systems composed of the same molecules as life, they cannot use matter and energy from the external environment to build internal order in the same way that life does. Thus, if we regard the concept of self-sustaining system, viruses should not be considered living entities (Moreira and López-García, 2009). Nevertheless, the fact that viruses contain a genome that encodes proteins following the same rules as cells –together with the reality that they can reproduce and evolve– are sufficient arguments for other scientists to consider them as life (Hegde et al., 2009; Navas-Castillo, 2009; Forterre, 2016; Harris and Hill, 2020). To further complicate the question, some recently discovered viruses have genomes and dimensions comparable to some cellular microorganisms (Van Etten and Meints, 1999; La Scola et al., 2003; Abergel and Claverie, 2020) and can even be infected by other viral entities called virophages (Fischer, 2011; Mougari et al., 2019).

If we go back to the times when life was taking its first steps, the boundaries between life and non-life were more blurred than they are today (Szostak, 2012). Although most life definitions contain both a thermodynamic and an inheritance aspect, it is easy to imagine that at primitive times there were entities in which these two properties were not present in the same way they are in modern life. An example of these gray zones would be represented by autocatalytic networks, in which all molecules can be synthesized through the reactions catalyzed within the set (Vitas and Dobovišek, 2019), giving rise to a self-maintained system. In these networks, information is not stored in homopolymers such as DNA or RNA and, therefore, evolution would reside in variations in inter-molecular interactions or in the mechanisms of obtaining energy from the environment. Viruses would be another example of gray zone that can exist as an inert form, the virion, and an active form, the virus multiplying inside the cell. In their active form, viruses are self-organized systems that store and transmit information, and are able to maintain their organization despite changes in the environment. Although viruses always need to infect a cell to reproduce, all the information necessary to manipulate the cell metabolism and produce a viral progeny is contained in the virus genome itself, which brings them closer to the definition of life. Forterre introduced the concept of virocell (Forterre, 2011) as a living system corresponding to any cell infected by a virus, whose main function is to produce new virions that would act as seeds or spores.

Whether viruses are considered living things or not, there is no doubt that finding viruses on a planet other than Earth would immediately make us think about the possibility of the existence of life on it. That life could be well established, taking its first steps, or even be extinct, having left viruses as the last vestiges of its existence. Viruses are inseparable companions of life as we know it. As we will discuss extensively in this review, viruses are probably very ancient and were present in the pool of genetic elements that gave rise to life (Koonin et al., 2006; Forterre and Prangishvili, 2009; Villarreal and Witzany, 2010; Durzyńska and Goździcka-Józefiak, 2015; Krupovic et al., 2019). Since that time, they have influenced, and continue influencing, evolution and distribution of life (Filée et al., 2003; Forterre, 2006; Koonin et al., 2006; Koonin, 2016). And there is no doubt of their relevance to conform the properties of the life that will populate our planet in the future.

Traditionally, viruses have been seen as disease-causing agents, a view that has changed radically in recent years. Pathogenic viruses are only a small fraction of the wide variety of viruses that exist. Many of them coexist peacefully with their hosts, sometimes even causing benefits. Often, advantages provided by viruses derive from their high capacity to modify and exchange pieces of their genomes, which convert them in great inventors of genes that, thanks to horizontal gene transfer, can be subsequently transferred to the cellular world (Muniesa et al., 2011; Penadés et al., 2015; Gilbert and Cordaux, 2017; Weiss, 2017; Irwin et al., 2022), giving rise to many of the intricate connections among the different branches of the phylogenetic tree of life.

Viruses exist practically everywhere in our planet, including extreme environments (Säwström et al., 2008; Yoshida-Takashima et al., 2012; Munson-McGee et al., 2020), which in some cases have physicochemical conditions that resemble some of those present in extraterrestrial environments. They are the most abundant biological entities on Earth and it is believed that all cellular organisms can be infected by some kind of virus (Suttle, 2005; Mushegian, 2020), which raises the question of whether they are an inevitable consequence of the emergence of living systems (Iranzo et al., 2016; Koonin et al., 2017) and must be present in any potential biosphere. Viruses, in the virion form, can withstand more extreme conditions than most cellular life. For this reason, sometimes they have even been considered as possible containers for the transport of genetic material between planets (Griffin, 2013; Berliner et al., 2018).

Finally, the rapidity of viral evolution is being exploited in multiple laboratories to carry out studies devoted to the research of biological evolution in real time (Elena et al., 2008; Kawecki et al., 2012; McDonald, 2019), which allows to establish some of the general principles governing this process. The importance in evolution of the error rate value, the intensity of selective pressures, the population size or the standing genetic diversity are just some examples of the topics that can be studied using viruses as experimental system.

The conclusion that arises from all the considerations described above is that, although viruses are not considered *sensu stricto* life, both current and past life in our planet has been shaped thanks to their action. As knowledge has advanced, viruses have gone from being considered enemies of life to being recognized highly relevant in fields as diverse as molecular ecology, evolutionary biology, structural biology or geomicrobiology. The same is happening with astrobiology, which is increasingly realizing that to understand life in a wide sense it is absolutely necessary to include viruses in its scenario (Griffin, 2013; Berliner et al., 2018).

## What can viruses teach us about the origin of life?

How old are viruses? Does their simplicity mean that they are intermediate forms between inert and living matter? Or do they represent an alternative path followed by the same precursors that gave rise to life? Some proposals also state that viruses are modern entities that have derived from cells. So far, we do not have a definite answer for these questions, although most of the evidence indicates that viruses, particularly those with an RNA genome, are very old. Therefore, their study can provide relevant information about the first steps toward life.

### Origin of viruses

There are three main hypotheses to explain the origin of viruses (Forterre, 2006; Koonin et al., 2006; Forterre and Prangishvili, 2009; Krupovic et al., 2019) that are briefly described below. Each of them has its implications in the relationships that the viral world establishes with the cellular world and in their mutual influence throughout life history. The three hypotheses are not exclusive in the sense that different groups of viruses could have been originated through different routes and at different times. For example, viruses with a DNA genome could not have existed until the emergence of this molecule, which places their origin at a later time than that of RNA viruses.

#### Escape hypothesis

Viruses are genetic cell elements that escaped from cells, becoming infectious entities unable to replicate on their own. According to this idea, prior to the emergence of DNA cells, there was a stage in the evolution of life that was dominated by RNA cells containing fragmented genomes (Woese, 1987, 2002). In contrast to the current world –in which the processes of genomic replication and cell division are perfectly integrated– in the primitive world, there were probably no mechanisms that regulated this integration in a precise way. Thus, it would be easier at that time that a genomic fragment could be released from a cell and become an infectious unit capable of replicating at the expense of the resources produced by others. For this hypothesis to be true, there should be more similarities between the genes of viruses that infect a particular domain of life and the cellular genes of that same domain. However, when virus protein structural domains have been analyzed, in many cases they are found to be similar in bacteriophages, archaeal viruses and eukaryotic viruses (Bamford, 2003; Benson et al., 2004; Rice et al., 2004; Trus et al., 2004; Krupovic and Bamford, 2008; Abrescia et al., 2012; Brum et al., 2013).

#### Reductive hypothesis

According to this scenario, viruses are the product of the degeneration of ancestral cells that lost their machinery for protein synthesis and energy production. These deteriorated cells became parasites of others that kept all their capacities. No evolutionary intermediary between viruses and cells has been found to date. Moreover, when viruses are compared with other parasites, the latter always retain some characteristics of their free-living time. The discovery of giant viruses that infect protists, and that sometimes possess genes of the protein translation machinery, triggered a resurgence of the reductive hypothesis (Schulz et al., 2017; Abrahão et al., 2018; Rodrigues et al., 2020). However, it is now widely accepted that these genes have been acquired from the host (Koonin and Yutin, 2018; Brahim Belhaouari et al., 2022).

#### Virus-first hypothesis

This hypothesis states that viruses, particularly those with RNA genomes, descend from the first molecules with replicative capacity that likely existed on Earth before the appearance of cellular life. For a time, this thinking received little consideration, because actual viruses always need a cell to replicate and, therefore, cells should have preceded viruses. However, primitive viruses were probably very different from today’s viruses. They could have been mere genetic parasites that emerged in replicator networks endowed with catalytic capacities. Parasites would multiply at the expense of the products of the network without contributing to their generation, as it is shown in some theoretical studies (Iranzo et al., 2016; Koonin et al., 2017). Additional, indirect support for this hypothesis comes from the fact that viruses use a great diversity of molecules [single-stranded RNA (ssRNA), double-stranded RNA (dsRNA), single-stranded DNA (ssDNA), and double-stranded DNA (dsDNA)] and strategies to store and replicate their genetic information, all of which brings to mind a time when different ways of preserving and processing genetic information were being tested (Koonin et al., 2006; Holmes, 2011b; Krupovic et al., 2019).

### The RNA world

How was the pre-cellular state of biological evolution where viruses could first emerge? In modern life, functional proteins can be synthesized because DNA stores information about the order in which amino acids should be linked together. But going from the DNA sequence to the sequence of a protein is an intricate process, which in turn requires the intervention of other proteins and complex structures such as ribosomes. Therefore, separation of information and function into two different molecules poses a paradox that can only be resolved if, in primitive life, information and function resided in the same molecule. Many scientists accept that at some stage of the evolution of life this molecule was RNA, although this does not preclude the existence of pre-RNA worlds in which catalysis and information could reside in other molecules that were more stable or easier to assemble than RNA. In this context, PNAs (peptide nucleic acids), which are synthetic polymers with a simple, achiral chemical structure composed of repeating N-(2-aminoethyl)-glycine units linked by peptide bonds (Nielsen, 2007) have received much consideration.

That RNA can be used to store hereditary information is demonstrated by the existence of viroids and RNA viruses. Due to internal base pairing, RNA molecules fold into secondary and tertiary structures that maximize their stability. As it happens with proteins in the current world, these structures can lead to the formation of active centers that facilitate the catalysis of certain chemical reactions. RNA molecules with catalytic capacity are called ribozymes. Their existence was first demonstrated in the eighties (Kruger et al., 1982; Bass and Cech, 1984; Altman, 1989) and confirmed in many subsequent studies (Chapman and Szostak, 1994; Hager et al., 1996; Lee and Lee, 2017; Janzen et al., 2020).

Everything described above led to postulate the existence of a hypothetical RNA world, made up of ensembles of molecules, the so-called primitive replicators, capable of storing and transmitting information (Gilbert, 1986; Oro et al., 1990; Sankaran, 2016). The catalytic properties of these molecules could have facilitated the emergence of a simple metabolism, which, once individualized in a compartment, would have been the basis for the appearance of the first cells (Koonin et al., 2006; Koonin, 2014; Szostak, 2017; Joyce and Szostak, 2018) and probably of the first parasites.

**FIGURE 1:**
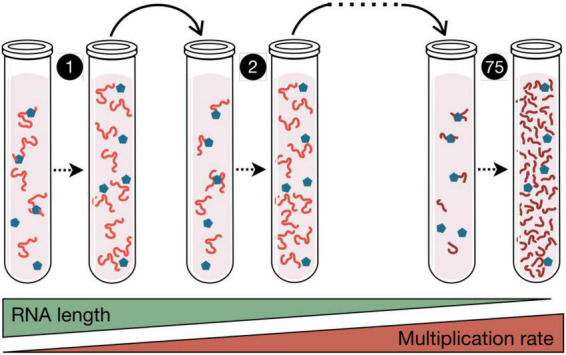
Spigelman’s Monster. In this experiment published in 1967, the RNA from phage Qβ (red lines) and the enzyme responsible for its copy (blue pentagons) were incubated in the presence of nucleotides, and subjected to *in vitro* replication. The products of each polymerization reaction were serially transferred to new tubes. After 75 of these transfers, 83% of the RNA genome was eliminated, which in turn increased the multiplication rate of the RNA population. This seminal paper by Mills et al. (1967) demonstrated that darwinian evolution can occur in a test tube. Similar serial transfers experiments are used in the study of virus evolution.

The first experiment demonstrating the capacity of RNA molecules to evolve in response to the environment was carried out by Sol Spiegelman in 1967 (Mills et al., 1967), using the bacteriophage Qβ, a virus with an RNA genome (Figure 1). The experiment carried out by Spiegelman consisted in mixing in a test tube a small amount of viral RNA with all the components necessary for its replication. This mixture was incubated under optimal conditions for the time necessary for the RNA to be copied. Then, a fraction of the molecules produced were transferred to a new tube containing fresh substrates for replication. This process of serial transfers was repeated over time, with the aim of finding out what happened to the RNA molecules. The result was that the virus genome lost a large part of its sequence, which allowed it to increase the copying rate (Figure 1). In new experiments it was found that, if the environment changed, the RNA molecules were capable of specific adaptations, increasing their replication speed under conditions such as imbalanced nucleotide concentrations or presence of ribonucleases (Levisohn and Spiegelman, 1969; Kacian et al., 1972; Biebricher and Gardiner, 1997). These experiments were highly relevant to demonstrate that molecules are also susceptible of optimization through darwinian evolution, a concept of crucial importance for the idea that before cellular life there must have been a phase of molecular evolution.

Theoretical studies inspired in Spiegelman’s experiments showed that error-prone replication combined with natural selection, and acting for a sufficiently long time in an infinite population of replicators (RNA-like molecules) differing in their fitness values, would result in the generation of a steady-state distribution of mutants in which each of them has a constant frequency (Eigen, 1971; Eigen and Schuster, 1979). This population structure was referred to as quasispecies, and the framework describing its dynamics quasispecies theory (Biebricher and Eigen, 2006). The major achievement of quasispecies theory was to describe mathematically a system involving multiplication of molecules with a regular production of error copies. The sequence displaying the highest replication rate within the quasispecies was denoted master sequence and corresponded to that most represented in the whole set of mutants, also known as mutant spectrum. The ensemble of all possible variations that can be generated from a genetic sequence is known as the sequence space. For a replicator of length *l* the sequence space is 4*^l^*, a number that can be enormous, even for short replicators. Quasispecies population structure was an important component of the catalytic hypercycle, a primitive model of organization of self-replicating molecules connected in a cyclic and autocatalytic fashion (Eigen and Schuster, 1979).

In addition to defining in quantitative terms a system of replicators that are copied with high error rate, quasispecies theory established the value of the error rate (the so-called error threshold) that was compatible with the conservation of genetic information (Biebricher and Eigen, 2005). When the error threshold is exceeded, the superiority of the master sequence disappears, all possible sequences become equally probable and the genetic information is lost.

Almost in parallel, experimental studies carried out with bacteriophage Qβ (Batschelet et al., 1976; Domingo et al., 1978) showed that this virus replicated with very high error rate and showed a great heterogeneity in its populations. The typical error rates of RNA viruses are on the order of 10^–6^ to 10^–4^ errors per nucleotide copied (Holland et al., 1982; Duffy et al., 2008; Sanjuán et al., 2010; Domingo et al., 2021). For comparison, error rate values (expressed with the same units as above) are around 10^–6^ for ssDNA viruses and in the ranges between 10^–8^ and 10^–7^ for dsDNA viruses, 10^–10^ and 10^–9^ for bacteria, and 10^–11^ and 10^–10^ for eukaryotes [(Gago et al., 2009) and references therein]. These high error rate values, together with the usually large sizes of viral populations, lead to the generation of highly diverse populations whose behavior could mimic that of the ensembles of replicators present in the RNA world and described in quasispecies theory. Nevertheless, factors such as the mechanism of genome replication, the variability and intensity of the selective pressures or the frequency of population bottlenecks are also highly relevant to determine the structure of a virus population and its evolutionary dynamics. Consequently, the substitution rate per nucleotide and year in some eukaryotic ssDNA viruses is similar to that of ssRNA viruses with the same genome length (Duffy et al., 2008).

### Viruses in the RNA world

If viruses are considered as simple genetic parasites, it is easy to think that their predecessors could have been present in the ensemble of primitive replicators that made up the RNA or the pre-RNA world. Their emergence would require the generation of mutants capable of being copied without contributing to the generation of the resources necessary for the copying process. The occurrence of these mutants would be relatively frequent, given the low fidelity of primitive replication (Krakauer and Sasaki, 2002). However, since parasites are not capable of a self-copying process, their permanence would require that they remain stable in the environment for long periods of time. This means that resistance to damage caused by external physicochemical variables probably constituted a relevant trait subjected to the action of natural selection in the RNA world, which later could have led to the emergence of viral capsids. The current existence of defective virus mutants−which lack the ability to encode all the proteins needed to complete their infectious cycle, but can give rise to a progeny when these proteins are provided by other viruses−corroborates the ease of the emergence of this kind of parasites (Huang, 1973; Cole, 1975; Grande-Pérez et al., 2005).

A valid approach to investigate whether viruses could originate in the RNA world is to examine the genes that encode specific viral functions, such as genome replication. In this case, there are two activities that are only found in the viral world and, more specifically, in RNA viruses. These are the RNA-dependent RNA polymerase (RdRp), and the reverse transcriptase (RT). In the cellular world, the RdRp and the RT perform very specialized functions, but are not involved in genome replication. It is striking that both enzymes share with DNA-dependent DNA polymerases a structural folding located in the main catalytic domain (Iyer et al., 2005; Kazlauskas et al., 2016), which seems to correspond to an ancestral RNA recognition motif (RRM) that was probably crucial for replication of both RNA and DNA (Cléry et al., 2008), serving as a cofactor for ribozymes in the RNA world. Other virus genes that also contain the RRM are the rolling-circle replication endonucleases, a superfamily of helicases, and some protein-primed DNA polymerases.

A relevant question is whether the primitive genetic parasites also encoded the proteins necessary for the formation of capsids. The current view is that virus capsid proteins evolved from cellular proteins at different stages of evolution (Krupovic et al., 2019). The high prevalence of some structural domains, such as the single jelly roll (a β-barrel fold consisting of eight antiparallel β-strands organized in two sheets that form the opposite sides of the barrel) or the double jelly roll in the capsids of viruses infecting organisms belonging to the three domains of life is probably due to their antiquity (Abrescia et al., 2012; Yutin et al., 2018; Santos-Pérez et al., 2019). In contrast to this, other capsid proteins may have been acquired more recently.

In conclusion, there are multiple lines of evidence indicating that the primitive RNA world could gave rise to two parallel and interconnected paths that led to the emergence of the first cells and the first viruses. Since then, both worlds have evolved in close link, influencing each other and contributing to increase their diversity, as we will see in the following sections.

## Role of viruses on the evolution of life

In the last decades, it has been recognized that viruses are major players in the history of life. They influence cellular evolution in different ways: acting as selective pressures, behaving as vehicles for horizontal gene transfer, and creating new genes that provide evolutionary novelty.

### Viruses as selective pressure

Virus infections favor organisms that possess some type of defense mechanisms. In the prokaryotic world, these mechanisms include abortive infections, modification of the molecules used as receptors, or the selection of systems, such as restriction endonucleases or CRISPRs, that recognize the virus genetic material and degrade it (Gao et al., 2020; Hampton et al., 2020; Wang et al., 2020b). In turn, the appearance of microorganisms that better resist the infection exerts a selective pressure on the viral population that will favor viruses able to counteract the cellular defenses (Geoghegan and Holmes, 2018; Stanley and Maxwell, 2018; Koonin and Krupovic, 2020). The repetition of these cycles involves concerted changes in the pathogen and its host, an *arms race* that accelerates the evolution of both the virus and the host (Buckling and Brockhurst, 2012; Koskella and Brockhurst, 2014; Watson et al., 2021).

A similar scheme works for viruses infecting eukaryotic cells. In the particular case of vertebrates (Kaján et al., 2020), it has led to the selection of the adaptive immune system that, with a limited number of genes, is able to cope with a wide variety of antigens. It is believed that one of the possible causes of the emergence of this system has been the need to deal with highly changing agents such as viruses (Holmes, 2004). Sometimes viruses reduce their virulence in certain host species. In this way, they do not induce a strong immune response and can remain multiplying longer in the same host. When, on the one hand, a protective immune response to the virus is generated and, on the other hand, virulence is reduced, a pacific coexistence between the virus and its host can be reached (Barnard, 1984; Holmes and Duchêne, 2019). When one of these viruses that are well adapted to a particular animal species is able to infect the human population, a zoonosis occurs. The absence of immunity to the pathogen, together with the fact that the virulence has not been attenuated in the new host, can lead to the emergence of a new disease (French and Holmes, 2020). COVID-19, AIDS, or the pandemics caused by new variants of influenza virus are just some examples of diseases caused by zoonotic viruses. In the short term, the introduction of these or other viruses in the human population can be very negative, but in the long term they may contribute to make us stronger against new threats.

### Horizontal gene transfer in prokaryotes

Horizontal or lateral gene transfer allows innovations that arise in one group of organisms to be shared by a much larger set. Through transduction, both specialized and generalized, viruses are relevant agents of horizontal gene transfer in bacteria and archaea.

Specialized transduction takes places during the lysogenic cycle and implies that the virus, instead of destroying the cell, integrates its genome into the cell chromosome, being transferred to daughter cells by vertical transmission (Feiner et al., 2015). Most sequenced bacterial genomes contain at least one prophage (a phage genome integrated) and, in some cases, these elements constitute 10–20% of their total genetic material. Sometimes, after the lysogenic integration of a prophage, some signals occur that provoke its release, initiating a lytic cycle that will end with the production of new phages and the lysis of the bacterium. In the process, the phage frequently carries some cellular genes with it, which can be transferred to other bacteria in the course of new infections. Among the most frequently phage-transferred genes there are some that increase the virulence of the bacterium (Plunkett et al., 1999; Nishida et al., 2021), confer resistance to antibiotics (Gabashvili et al., 2020, 2022), help it to survive under difficult circumstances (Lindell et al., 2004, 2005; Sharon et al., 2007; Fridman et al., 2017), or allow the exploration of new ecological niches (Sullivan et al., 2005). In all cases, the advantages obtained from the integration of virus genetic material is what contributes to their persistence. Taking as an example the most abundant photosynthesizers on Earth –*Prochlorococcus* and *Synechococcus* (Flombaum et al., 2013)–, genes for the components of these cyanobacteria’s photosystems are also present in the genomes of their phages (Mann et al., 2003), which seems to have amplified the evolution and expansion of these systems. Furthermore, the expression of the viral photosynthetic genes during infection enhances the photosynthetic activity of the host (Fridman et al., 2017), which increases viral replication, especially under low-nutrient conditions (Hurwitz et al., 2013).

Generalized transduction occurs during the lytic cycle of some viruses that, at the beginning of the infection, cause cellular DNA breakage (Thierauf et al., 2009; Waddell et al., 2009). When the progeny viruses are assembled, it is frequent that they do not distinguish between their own genome and the fragments of the cellular genome. The foreign DNA can be introduced into the virus capsids and, thanks to homologous recombination mechanisms, become integrated in the genome of the new infected microorganism.

### Virus endogenization

The insertion of virus genomes into the cell chromosome is not exclusive to the prokaryotic world. When it happens in the chromosomes of the host reproductive cells of vertebrates and the inserted provirus is maintained over time, the process is called viral endogenization (Holmes, 2011a; Feschotte and Gilbert, 2012; Greenwood et al., 2018). After the initial insertion event, the provirus can spread in the cellular genome through a mechanism similar to that used by retrotransposons. During their dissemination, the viral sequences accumulate mutations or undergo epigenetic modifications that inactivate the expression of most of their genes (Wolf and Goff, 2008; Rowe and Trono, 2011; Yang et al., 2022), so that after a time they can no longer lead to the production of infectious viral particles.

The integrated viral elements can disrupt the sequence of genes or their regulatory elements, causing loss of function. Consequently, individuals carrying this kind of exogenous DNA are normally eliminated by natural selection. However, sometimes viral genomes persist through generations, expanding in the population until they become fixed. In these cases, the DNA of viral origin becomes host DNA. The great number of viral insertions that have been fixed in vertebrate lineages (Hayward et al., 2015) represents a substantial source of genetic material that adds variability to genomes and that sometimes can provide benefits. For instance, the insertion of a provirus in a region near the pancreatic amylase gene has allowed this enzyme to be also expressed in saliva in humans, so that starch digestion can begin in the mouth (Ting et al., 1992). This is just an example of how virus regulatory sequences can modify gene expression in the host. In addition to the effects on individual genes, the process of provirus amplification can lead to the dispersion of viral regulatory elements throughout the cellular genome, producing large reorganizations of gene expression that can produce major evolutionary innovations (Feschotte, 2008; Koonin, 2016). The amplification process also facilitates the exchange of genetic material between different sequences of the genome, resulting in the loss of some regions, the duplication of others, or changes in their location (Hughes and Coffin, 2001; Bai et al., 2021).

Despite the serious consequences that the expression of virus genes usually has for the host, there are some situations in which their products have been recruited to perform cellular functions. A classic example is the expression of viral proteins that provide immunity to infection by related viruses (Arnaud et al., 2008). But perhaps the best example of genes from endogenous viruses that have allowed the emergence of novel functions are those encoding for syncytins. Syncytins are proteins that promote the fusion of a type of cells called trophoblasts to give rise to the formation of the syncytiotrophoblast, a cell layer that forms part of the placenta. Syncytin genes have their origin in a gene from retroviruses, specifically in the gene env, which has fusogenic activity (Dupressoir et al., 2011). It appears that the event of domestication of env genes of viral origin has occurred at least six independent times throughout evolution, in different mammalian species, including humans (Rawn and Cross, 2008).

### Role of viruses in major evolutionary transitions

Darwinian theories explain evolution as the result of the progressive accumulation of heritable changes of small effect. However, these small changes cannot explain the increases in biological complexity associated with some evolutionary innovations that require drastic changes in the amount of genetic information and/or in the way it is processed. When these innovations involve the integration of entities from a lower level of organization into a higher one that comes to constitute a new level of selection, we speak of major evolutionary transitions (Szathmáry and Smith, 1995; Szathmáry, 2015). The continuous arms-race between viruses and their hosts, together with the frequent exchange of genetic material driven by viruses, has frequently contributed to the increase of biological complexity (Forterre, 2006; Koonin, 2016; Mizuuchi et al., 2022), as illustrated in some of the examples given in the previous section.

It is believed that the need to defend against primitive viruses promoted that the molecules carrying genetic information became grouped in compartments that, in addition to create barriers for the spread of parasites, facilitated the cooperation between non-parasitic replicators, contributing to stabilize the whole system, which emerged as a new selection unit (Szathmáry and Demeter, 1987; Higgs and Lehman, 2015). Within compartments, selection for increased genome size would also have been generally favored, as a mechanism to fix the most favorable genetic combinations. Primitive compartments could be protective micro-environments in mineral surfaces or simple lipid vesicles.

Mathematical modeling of replicator systems also suggests that resistance to parasites increases when information and function are separated in different molecules. It is easy to understand the advantages of this fact, since parasites could only take advantage of the functional molecules and not of the informative ones, which would facilitate their survival. However, we still do not know much about how DNA replaced RNA as an information storage molecule and about the appearance of the first protein catalysts, which probably coexisted for some time with RNA or RNA-like catalysts. The presence in retroviruses of reverse transcriptase activity, which converts viral RNA to DNA, has led some authors (Forterre, 2006) to think that this enzyme could be involved in the replacement of RNA for DNA as informative molecule, but this is something that with the current evidence cannot be affirmed with certainty.

Viruses may also have played a role in the origin of eukaryotic cells, which possess mitochondria and an internal membrane system that delimits a nucleus in which the genomic DNA is located. Mitochondria have their origin in a process of endosymbiosis between a bacterium and another cell, probably an archaeon. It is intriguing that in all them, the bacterial RNA polymerase has been replaced by a phage RNA polymerase (Filée and Forterre, 2005), although it is difficult to elucidate the advantages provided by this fact. The eukaryotic nucleus allows the uncoupling between transcription and translation. The discovery of viral factories (Fridmann-Sirkis et al., 2016; Chaikeeratisak et al., 2017), showing that some bacterial and eukaryotic viruses can establish a similar separation, led to propose that the eukaryotic nucleus has a viral origin (Koonin, 2015; Bell, 2020; Takemura, 2020). However, this idea is not exempt of controversy and there are other hypotheses that state that the nucleus originated from invaginations of the plasmatic membrane in an ancestral prokaryote or has a symbiotic origin without virus participation.

## Viruses at the planetary scale: The virosphere

The ongoing advances in high-throughput sequencing technologies and metagenomic analyses have been crucial to reveal the immense diversity and pervasive distribution of viruses throughout our planet (Zhang et al., 2018). As previously indicated, viruses seem to have colonized –presumably associated with cellular hosts– every place where life can be sustained, while exploring vast areas of the evolutionary sequence space. The viral composition of the Earth’s biosphere is known as the virosphere.

### The immensity of the virosphere and its diversity

The abundance of viruses in aquatic and terrestrial environments has been estimated to be 10–100-fold higher than that of unicellular organisms, although this ratio is dynamic and varies across ecological niches (Wigington et al., 2016). A milliliter of seawater can contain up to hundreds of millions of viruses (Bergh et al., 1989), and up to a billion of them can be found in a single gram of soil (Swanson et al., 2009). Thus, it has been estimated that the total number of viral particles in the planet is in the astronomical order of 10^31^ (Hendrix et al., 1999; Mushegian, 2020), outnumbering all cellular organisms.

Metagenomic studies aimed at unveiling the viral makeup of different environments around the globe have barely scratched the surface of the virosphere’s diversity. A remarkable effort is the deep sequencing of samples collected during the Tara Oceans expeditions, in which nearly 35,000 samples from the ocean were collected between 2009 and 2013 in a ship transect around the globe (Brum et al., 2015; Roux et al., 2016; Gregory et al., 2019; Sunagawa et al., 2020). This data set consisted mostly of viruses with dsDNA genomes (excluding large portions of the virosphere), and yet, they are taxonomically more abundant than bacteria, archaea and micro-eukaryotes together (Abreu et al., 2022).

The rate of discovery of RNA viruses has lagged behind that of DNA viruses, mostly due to the technical requirement of converting RNA to DNA prior to sequencing. However, thanks to advances in metatranscriptomics, there has been a recent explosion in the amount of RNA virus data. The collection of RNA viruses in the databases doubled in 2016 after the metatranscriptomic analysis of numerous invertebrates (Shi et al., 2016), and doubled again in 2020 after sequencing just 10 L of sea water (Wolf et al., 2020). In 2022, by mining data from databases, the amount of known RNA virus sequences increased fivefold with the description of ∼330,000 novel RNA viruses (Neri et al., 2022), in addition to the discovery of ∼130,000 highly divergent RdRp sequences from novel viruses (Edgar et al., 2022). In each of these RNA virus data expansions, novel virus clades have been revealed. For instance, the exploration of the RNA virus oceanic composition has reached enough depth to reveal a novel group of capsid-less viruses, the *taraviricots*, which provides a missing link between retroelements and RNA viruses. The lineage of the taraviricots seems to predate the split between RdRPs and retrotranscriptases, suggesting that virus-like replicators encoding an RdRp are ancestral to RNA viruses and retroviruses, and probably derived from the primordial pool of replicators of the RNA world (Zayed et al., 2022).

### Organization of the virosphere and its challenges

**FIGURE 2:**
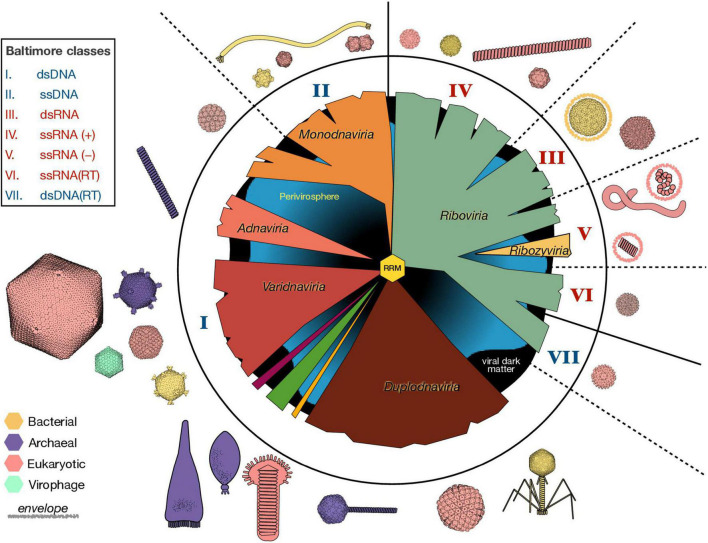
Organization and Diversity of the Virosphere. The vast genetic diversification of viruses is reflected in a variety of viral genome lengths (1.8 Kb–2.5 Mb) and organizations (e.g., circular vs. linear, segmented or not), virion sizes (17 nm–1 μm) and morphologies, host ranges, and in the types of interactions between viruses and their hosts. Here, we represent the six virus realms in the middle circle, as they emerge from an ancestral RNA-recognition motif (RRM) in the center. The roman numbers indicate the nature of the packaged nucleic acid according to the Baltimore classification scheme, where blue numbers represent DNA and red numbers RNA. Non-viral mobile genetic elements (perivirosphere) and the unknown viral sequences (viral dark matter) are included in the background. Some representative virus morphologies from viruses infecting all three domains are depicted with approximate relative sizes and colored according to their host. The available molecular structures were downloaded from RCSB Protein Data Bank and visualized with UCSF ChimeraX. ss, single-stranded; ds, double-stranded; RT, retro-transcriptase.

Considering the colossal diversity of viruses, classifying the virosphere is not a trivial task. For decades, the main virus classification scheme was based on the nature of the packaged nucleic acid and traits such as virion morphology, replication strategy, host organism and type of disease. Given the explosion of virus sequence information fueled by metagenomics and for which no phenotypic information is available, these criteria seem no longer practical. The 7 Baltimore classes provided an organization framework based on the nature of the packaged nucleic acid and the strategies by which mRNAs get produced from these DNA or RNA genomes (Figure 2; Baltimore, 1971). However, the Baltimore classification does not accurately represent the evolutionary relationships among viral lineages.

A phylogenetic approach to virus classification is difficult, due to the absence of a common marker for virus classification. Moreover, viral genomes are modular and virus evolution is greatly shaped by lateral gene transfer. Despite these difficulties, by using phylogenetic analyses of virus hallmark genes and gene sharing networks, a new “megataxonomy” of viruses has recently been established (Koonin et al., 2020). In this new classification of viruses, a new taxonomic rank –the realm– had to be defined to distinguish between virus taxa with no common ancestry. The six virus realms are: (i) *Riboviria*, which contains all RNA viruses and retroviruses, and that may have derived from the primordial pool of replicons, (ii) *Monodnaviria*, with viruses whose DNA genomes (most single stranded) encode an endonuclease for replication, (iii) *Varidnaviria*, dsDNA viruses with vertical jelly roll capsid proteins, (iv) *Duplodnaviria*, dsDNA viruses with HK97-fold capsid proteins, (v) *Adnaviria*, which helical virions containing A-form DNA genomes, and (vi) *Ribozyviria*, whose members have circular RNA genomes similar to viroids (Figure 2). Although different viruses probably emerged at different times and through different routes, all viruses in these six realms use replication proteins derived from the ancestral RNA-recognition motif (RRM), that probably has its origin in the RNA world (see section “Viruses in the RNA world”) and subsequently predated cellular life (Koonin et al., 2020). This new classification scheme has been recently ratified by the International Committee on Taxonomy of Viruses.

The six virus realms currently contain more than 200 viral families. However, the detection of viral sequences with no recognizable homologs is far from uncommon, and a vast portion of sequence data remain unclassified. Current advances in sequence analysis are key to disentangle this “viral dark matter” (Krishnamurthy and Wang, 2017). Another challenge to virus classification is the existence of viral chimeras. While recombination between viral genomes within viral groups is frequent (Pérez-Losada et al., 2015), gene sharing extends beyond the borders between the virus realms. The cruciviruses, for example, is a group of chimeric viruses whose circular ssDNA genomes encode a capsid protein also found in RNA viruses (de la Higuera et al., 2020).

The virosphere also include mobile genetic elements that are not bona fide viruses, such as viroids, plasmids and retroelements, which have been relegated to the “perivirosphere” (Koonin et al., 2021). The delimitation of the perivirosphere, however, is blurry, as mobile genetic elements can acquire capsid genes to become viruses (Krupovic et al., 2019; Koonin et al., 2022). For instance, ssDNA viruses belonging to the realm Monodnaviria appear to have emerged multiple times from plasmids by capturing capsid genes from RNA viruses (Kazlauskas et al., 2019).

### Impact of viruses on Earth’s biogeochemical cycles and organismal biodiversity

Microbes are the main drivers of the biogeochemical cycles that define ecosystems, but they also are susceptible to virus infection, e.g., it is estimated that ∼20% of the bacteria in the oceans are lysed by viruses per day (Suttle, 1994). These ongoing viral infections shape microbial community structures and are necessary for nutrient cycling (Dominguez-Huerta et al., 2022).

Viruses can impact food webs and biogeochemical cycles in different ways. Cell death caused by lytic viruses leads to the release of dissolved organic matter, which is then available for its consumption by low trophic levels. This process, called “viral shunt,” allows nutrients to be reused by other microorganisms rather than getting passed on to higher trophic levels (Wilhelm and Suttle, 1999; Zimmerman et al., 2020). The released organic matter from viral lysis can be particularly rich in nucleotides and amino acids (Ankrah et al., 2014). Thus, the viral shunt is important for the cycling of not just carbon, but other nutrients such as nitrogen (Shelford et al., 2012), phosphorus, and iron (Gobler et al., 1997; Poorvin et al., 2004). A complementary process, the “viral shuttle,” describes how viruses help the export of carbon by sinking organic matter to the bottom of the ocean (Guidi et al., 2016; Sullivan et al., 2017). The virus shuttle has been observed in algal blooms –often terminated through virus infection (Brussaard, 2004)–, in which the production of virally induced polymeric matrices enhances the sink of cellular aggregates to the sea floor (Laber et al., 2018). Virus composition have proven to be a great predictor of carbon flux in aquatic (Guidi et al., 2016; Kaneko et al., 2021) and terrestrial ecosystems (Emerson et al., 2018; Trubl et al., 2018; Starr et al., 2019), which further demonstrates the importance of virus infections in biogeochemical cycles.

The lysis of cells upon infection can have a significant effect on top-to-bottom control on biogeochemical cycles. However, viruses do not need to kill their host to impact ecosystems. During infection, viruses reprogram the cellular metabolism to divert resources to progeny production, which at times involves the expression of virally encoded metabolic genes that were likely acquired from cells (see section “Horizontal gene transfer in prokaryotes”) (Howard-Varona et al., 2020). Since a significant portion of the Earth’s microbiome is infected at any given time, these viral “auxiliary metabolic genes” may carry functions that can have an impact on planetary processes (Zimmerman et al., 2020). The analysis of auxiliary metabolic genes from virus genomic data can be used as a proxy to understand the impact that viruses exert on ecosystems, including the control of sulfur and nitrogen cycling in the ocean (Roux et al., 2016).

Viruses are also community structure regulators. They can propagate more effectively when their host is in abundance, thus, according to the “kill-the-winner” hypothesis, lytic viruses help maintain a balance and promote diversity in microbial communities (Thingstad and Lignell, 1997). This seems the case in the collapse of algal blooms by viral infection (Suttle, 2007). However, there are other examples where a particular species seems to be pervious to viral infection and become dominant (Yooseph et al., 2010). This scenario, called “king-of-the-mountain” (Giovannoni et al., 2013), suggests that high levels of recombination in these organisms allow the rapid adaptation of defense systems against their viruses (Zhao et al., 2013). This positive feedback loop in the co-evolution of virus and host is another example of the arms race described in section “Viruses as selective pressure,” by which viruses fuel organismal evolution and diversification (Giovannoni et al., 2013).

The realization of the important roles that viruses play in Earth’s habitats underscore how pertinent it is to consider genetic parasites in the study of biological systems. For that matter, it is relevant to also look at those ecological niches where life defies its own physicochemical boundaries.

### Viruses in extreme environments

The study of life in harsh conditions is essential to astrobiology. On one hand, extremophilic organisms shed light on the limits of habitability, i.e., the range of environments capable of supporting life in terms of temperature, pH, salinity, pressure, humidity and radiation (An Astrobiology Strategy for the Search for Life in the Universe, 2019). On the other hand, the biology of extreme environments can help to understand the conditions for life emergence (Baaske et al., 2007; Morowitz and Smith, 2007; Weiss et al., 2016).

Extreme environments are no exception to the wide distribution of viruses. They can thrive at temperature ranges between −12 and 96°C (Munson-Mcgee et al., 2018), pH values from 1 to 11 (at the least), saturated salinity (Pietilä et al., 2013), and extreme dryness (Gil et al., 2021). According to this, viruses have been found in hydrothermal vents (Russell and Hall, 1997), including those located at great depths in the oceans (Thomas et al., 2021), in oil fields (Zheng et al., 2020), in the soils of hyper-arid deserts (Zablocki et al., 2015), at hundreds of meters in the subsurface (Daly et al., 2019), or in places with salt concentrations far above the optimal for most living beings (Santos et al., 2012). Many of these locations share environmental characteristics that also occur in extraterrestrial environments. For example, submarine hydrothermal vents could be considered a terrestrial analog of those postulated to exist at the bottom of the inner oceans of the icy moons of some gas giants, such as Enceladus, Europa, or Ganymede. Oil field biology could help us to understand whether life could develop in the hydrocarbon lakes that exist on Titan, while the study of the subsurface could help us to figure out the possible life that existed (or still exists) on Mars. As we have already indicated, wherever there is cellular life there are also viruses, which reinforces the idea that the search for these entities could be used as a proxy for the search for life beyond Earth, something that is not receiving all the consideration it deserves.

Like in other ecosystems, extreme environment viruses can encode auxiliary metabolic genes that may be transferred between organisms. In hydrothermal vents, these genes are involved in metabolic pathways that are critical for survival in this environment including –but not limited to– sulfur-oxidizing enzymes (Anantharaman et al., 2014; Castelán-Sánchez et al., 2019). Viruses can also increase the adaptability of multicellular organisms to extreme environments. The triple-symbiosis in the thermotolerant terrestrial plant *Curvularia protuberata* is a classic example of how a virus infection –*via* fungal interaction in this case– can provide tolerance to heat stress (Márquez et al., 2007).

Archaea are typically found in the whole spectrum of extreme environments, and their viruses (archaeoviruses) –given the striking diversity of their unique morphologies (Figure 2)– are one of the most captivating types of extremophilic viruses. The virions of archaeal viruses have shapes suggestive of lemons (*Fuselloviridae*), bottles (*Ampullaviridae*), droplets (*Guttaviridae*), or spirals (*Spiraviridae*) (Figure 2; Baquero et al., 2020). Most archaeoviral families encode proteins with no detectable homologs in other viral groups, which hinders their assignment into any of the established realms of the virosphere.

The molecular adaptions of viruses to withstand the extreme extracellular conditions in these habitats remain underexplored, but structural studies have revealed special features in virions isolated from these locations. Some archaeoviruses package their genomes in the A-form of DNA (DiMaio et al., 2015). This DNA conformation is more compact than the more usual B-form, suggesting that this DNA stabilization mechanism is an adaptation to extreme conditions (Wang et al., 2020a). Another archaeovirus, *Aeropyrum coil-shaped virus* (not only the largest known ssDNA virus, but also stable at 95°C), forms a nucleoprotein with its circular ssDNA genome, which coils into an intertwining fiber that compacts adopting the shape of a spiral (Mochizuki et al., 2012). Archaeoviruses can also incorporate ether lipids from their extremophilic host in their virions, which seem to protect against chemical stress (Boyd et al., 2013). Archaeal lipids are also present in the structure of hyperthermophilic spindle-shaped viruses, but its location remains under debate (Quemin et al., 2015; Han and Yuan, 2022).

These structural adaptations to attain virion stability in extreme conditions highlight the ability of viruses to disperse within, but also beyond, the natural habitat of their hosts. Spindle-shaped archaeoviruses are found in a wide variety of extreme environments around the globe, including deep-sea hydrothermal vents, hypersaline environments, anoxic freshwaters, cold Antartic lakes, terrestrial hot springs and acidic mines (Wiedenheft et al., 2004; Krupovic et al., 2014), even though their hosts are highly adapted to isolated geographies (Whitaker et al., 2003). However, other studies show that viruses present in hydrothermal vents have restricted host range and are not widely distributed among vent sites (Thomas et al., 2021). All this raises questions about how viruses disperse at a planetary scale (Stedman et al., 2006), and possibly beyond (Griffin, 2013).

### Viral (inter?) planetary distribution

Viruses can disperse passively throughout different geographic scales in their stable virion form. According to the seed-bank hypothesis, viruses can be transported by oceanic or wind currents, and be recruited in ecosystems where they have an opportunity to thrive (Breitbart and Rohwer, 2005). Thus, viruses constitute a global genetic reservoir from which local virus communities reshape within biomes across ecological zones (Brum et al., 2015; Gregory et al., 2019).

Viruses disseminate in the atmosphere associated with dust particles or aerosolized liquid droplets. Therefore, vast amounts of viral particles can be globally spread from events such as desert storms or the release of sea spray (Baylor et al., 1977; Griffin et al., 2001). Some of these virions can be transported past the planetary boundary layer into the troposphere, further favoring long-range viral dispersal (Reche et al., 2018). The deposition of viruses from the troposphere has been observed for air masses coming from both the ocean and the desert, which reflects the magnitude of virus movement across the globe (Reche et al., 2018).

Given the potential of viruses to disperse at a planetary scale, it is likely that some of the viral particles escape the Earth. The same possibility could be applied to extraterrestrial virospheres. In addition to viral adaptations to extreme environments, viruses have been shown –in experimental setups– to survive high doses of UV radiation, ionizing radiation, X rays, high vacuum, desiccation, or microgravity conditions (Koike et al., 1992; Hegedüs et al., 2006; Horneck et al., 2010; Vaishampayan and Grohmann, 2019; Sharma and Curtis, 2022), a fact that would support their possible involvement in the transport of genetic material between planets. Particular consideration deserves the finding that under simulated hot spring conditions, viruses can fossilize upon the aggregation of silica deposits (Laidler and Stedman, 2010; Orange et al., 2011). Since virus silicification renders virions resistant to desiccation, it has been suggested as a potential long-range dispersal mechanism (Laidler et al., 2013). Although the ability of viruses to endure outer space conditions and disperse cosmically remains poorly understood, the field of astrobiology would benefit from considering viruses as potential carriers of life signatures (Griffin, 2013; Berliner et al., 2018).

## Viruses as biosignatures

The search for extraterrestrial life relies on the study of indicators of extant or past life. Biosignatures are defined as detectable substances, objects or patterns that are likely originated from life processes, and not abiotically (Chan et al., 2019). We could detect extraterrestrial life: (1) remotely, by the observation of astronomical bodies; (2) *in situ*, through space exploration, or (3) in transported samples. A good biosignature should be abundant, unlikely to originate in the absence of life, persistent in time (especially for the detection of past life or in transported samples), and easily detectable (An Astrobiology Strategy for the Search for Life in the Universe, 2019). The expectations of what life looks like in other planets is largely based on our understanding of our own biosphere. However, special attention needs to be put into universal and agnostic biosignatures that could apply to unknown forms of life.

The abundance and importance of viruses in Earth’s history and processes suggest that virus-like agents should be abundant members of other biospheres. Would these extraterrestrial molecular parasites form detectable structures like viruses in our planet do?

### Icosahedral capsids as agnostic biosignatures

**FIGURE 3:**
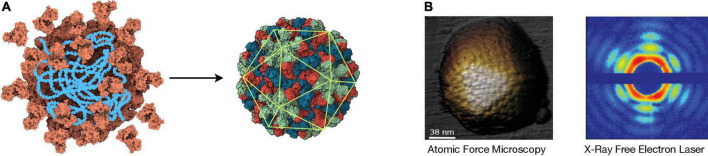
Icosahedral Symmetry Formation and Detection. **(A) Left:** Cartoon representing the assembly of identical capsid protein subunits (orange) around a nucleic acid (blue). **Right:** The resulting virion has icosahedral symmetry, as indicated by the overlapping icosahedron (green). The different colors represent the three different positions that the subunits can occupy in this *T* = 3 structure that corresponds to the plant-infecting RNA virus TBSV (pdb: 2TBV). In this case, a capsid is assembled from 180 copies of the capsid protein. The structural data were downloaded from RCSB Protein Data Bank and visualized with UCSF ChimeraX. **(B) Left:** Image of an adenovirus particle detected by atomic force microscopy (AFM) [adapted from de Pablo and San Martín (2022)]. **Right:** X-ray diffraction pattern caused by a mimivirus particle using X-ray free-electron laser (XFEL) [adapted from Ekeberg et al. (2015)].

To increase their stability and protect their genetic material, viruses coat their genomes with a protein capsid encoded in its own genome. However, at least in the life we know, it is impossible to translate a protein large enough to package its own code (i.e., the molecular weight of a codon is ∼1,000 g/mol and amino acids are 75–203 g/mol, making genetic information bulkier than its product). This size dilemma is overcome in viruses by using capsid proteins with the ability to self-assemble. Thus, a multimeric container can be formed from identical copies of a single gene product. In this case, these protein subunits have the exact chemical and spatial properties and must interact with each other in an equivalent or quasi-equivalent manner, which is achieved by the formation of symmetrical arrangements (Figure 3A; Caspar, 1956; Watson and Crick, 1956). Structural symmetry is a staple of the genetic economy characteristic of viruses, as it allows the creation of containers using the minimal amount of information.

Icosahedral symmetry is a prevalent virus architecture, as the spherical containers it provides are the most efficient in terms of volume-to-surface ratio. Capsids with icosahedral symmetry have emerged multiple times throughout the evolution of the virosphere (Krupovic and Koonin, 2017), and are present in viruses infecting all domains of life, and packaging any form of nucleic acid (Figure 2). The principle of quasiequivalence (Caspar and Klug, 1962) explains, by a simple rule of triangulation, how any multiple of 60 subunits can assemble into a wide spectrum of structures with icosahedral symmetry. Peptides as short as 24 residues have been shown self-assembly into icosahedral structures (Matsuura et al., 2010), suggesting that these apparently complex architectures could be possible even at early stages of life. Moreover, the icosahedral arrangement of capsid proteins is a direct consequence of a free energy minimization of the chemical interactions between subunits (Zandi et al., 2004), which allows the spontaneous assembly of highly stable structures (Garmann et al., 2019). The geometrical and thermodynamic constraints that pressure the selection of icosahedral symmetries for the formation of molecular containers are probably universal, and as such, in principle should be expected in extraterrestrial virospheres. However, constraints different from those operating in terrestrial life could be relevant in the architecture of the capsids of possible extraterrestrial viruses. When talking about life outside the Earth, it is necessary to take special care that the properties of what we know do not bias our expectations about what we can find. Even in terrestrial viruses, there are other types of capsids apart from the icosahedral ones (Figure 2). An example already cited is the wide variety of morphologies exhibited by archaeal viruses. Another problem that can arise is the detection of structures similar to viral capsids, but whose origin is due to chemical or geological processes. Distinguishing whether an apparently biological structure originates from biotic or abiotic processes is not easy, as has been shown on several occasions, including the controversial detection of microfossils in the ALH84001 meteorite.

Viral structures are good candidates for biosignatures as they are probably abundant components of other biospheres and –given their major role in protecting and dispersing information– can be stable in a wide range of environmental conditions. Despite the small size of viral particles, a plethora of techniques can be used to detect and analyze them. Among them, the most relevant to obtain meaningful structural information are electron microscopy, atomic force microscopy (AFM) and X-ray crystallography. Electron microscopy (cryo-EM in particular) is a powerful method broadly used for structure determination (Jiang and Tang, 2017). However, it requires complex sample manipulation and its throughput is still limited. AFM analyzes the contour of a sample by scanning it mechanically with an ultrafine probe, obtaining virus structure information at high resolution (Figure 3B). AFM technology is advancing at a fast pace, allowing the analysis of several mm^2^ per second at nanometer resolution (Marchesi et al., 2021). AFM devices can be very compact, which is a clear advantage for biosignature detection *in situ*, as they can be –and have been (Smith et al., 2008)– incorporated into spacecrafts. The usage of X-ray diffraction for the study of virus morphology has seemed to succumb to more convenient techniques such as cryo-EM; however, new advances are revolutionizing the field (Shoemaker and Ando, 2018).

The cutting edge of X-ray diffraction technology is the X-ray free-electron laser (XFEL). The XFEL produces coherent and ultra-bright X-ray femtoseconds-long pulses that can be applied to an aerosolized sample (Chapman et al., 2011). This not only allows for the observation of ultra-fast protein motion at near-atomic resolution (Pandey et al., 2020), it could –most importantly to our matter– scan a sample for molecular structures of interest at a great speed and depth. The XFEL offers a great flexibility in terms of temperature and sample conditions, and it can work under non-equilibrium while penetrating through matter without interfering with external fields (Meents and Wiedorn, 2019). Because icosahedral capsids are highly symmetrical, XFEL can produce clear diffraction patterns from single viral particles with no previous crystallization (Figure 3B; Seibert et al., 2011), which –coupled with machine learning (Suzuki et al., 2020)–, would offer great sensitivity for the detection of icosahedral symmetry in a complex sample. To our knowledge, however, there has not been any astrobiological research efforts in this direction. XFEL technology requires big structures to operate, which would only allow the analysis of samples transported back to Earth; however, technological improvements aimed at reducing the size of XFEL instrumentation are underway (Rosenzweig et al., 2020). Although abiotic molecules with icosahedral symmetry exist –e.g., fullerene and closo-carboranes–, these are much smaller than what is expected from biotic icosahedral structures and should be easily discriminated by the methodologies described above.

### Other viral signatures

Biosignatures based on the detection of chiral excesses, isotopic fractionation or the products of degradation of the biomolecules composing viruses can also be considered as possible proofs of their existence in extraterrestrial biospheres that may even be extinct. Carrying out experiments devoted to determining the chemical and morphological footprint left by viruses immobilized on mineral substrates, before and after being subjected to simulated space conditions in laboratory chambers, can be very useful to know what we can expect to find in another place of space where viruses have been present at some point in its history.

Additionally, there are biosignatures that are not necessarily virus-specific, but that would imply their detection in one way or another. For instance, the usage of chemical polymers to store and transmit information is a likely possibility in other life forms. Given the role of viruses as reservoirs and vehicles of genetic information on Earth and their abundance and dispersibility, there are better chances to detect virus-like information packages than more complex life-forms. There are procedures that allow the concentration and purification of viral particles (flocculation with polyethylene glycol and salt, or differential ultracentrifugation) that could be used to increase the probability of life detection in a sample. Sequences technologies based on electrochemistry (e.g., Nanopore) could then be used to detect information patterns from possible biopolymers in a sample, even if they are distinct from DNA or RNA (Rezzonico, 2014). Nanopore-based technology is very compact and, in synergy with other techniques, has successfully been used in samples from a Mars-analog environment, where DNA –including viral sequences– was detected at very low concentrations (Maggiori et al., 2020).

Other life detection methods that are being investigated include immunoassays using antibody microarrays for the recognition of a broad spectrum of common prokaryotic antigens (García-Descalzo et al., 2019). Virus hallmark motifs should be added to this collection of antigens. Antibodies for the detection of the jelly roll fold characteristic of many viral capsids or the ancestral reconstruction of the primitive RRM that antecedes most of viral and cellular replicases (Koonin et al., 2022) would be exceptional candidates for future versions of the LDChip [Life Detector Chip; (García-Descalzo et al., 2019)].

Additional roles of viruses in the detection of life have been discussed (Berliner et al., 2018), including the remote identification of virally provoked phenomena such as the formation of huge calcium carbonate deposits upon lytic termination of algal blooms described in section “Impact of viruses on Earth’s biogeochemical cycles and organismal biodiversity.” Viruses may have also played a role in the transformation of microbial communities, including the generation of microbial mats and stromatolites. If we take into account that the most ancient evidence of life on Earth correspond to stromatolites, the study of whether viruses can manipulate the microbial metabolism to influence carbonate precipitation and other processes related to the lithification of microbial communities can be highly relevant to interpret mineral biosignatures through geological time (White et al., 2021). Research efforts toward the study of viral biomarkers have been scarce, but increasing understanding of the role of viruses in origin and evolution of life will likely put viruses in the spot-light of astrobiological investigations in the near future.

## Viruses as a tool for determining general evolutionary principles

A fundamental topic in Astrobiology is the study of how life has diversified since its origin to give rise to all the forms in which it currently manifests. However, the study of the evolutionary process is not an easy task, because its primary cause -the generation of mutations- occurs by chance and is subjected to multiple contingencies that condition the action of selective processes. Moreover, its results usually require long periods of time, which makes this process difficult to observe in real time. A valid approach consists of analyzing how biological diversity has changed throughout life history and as a function of the physicochemical parameters of the environment. However, natural environments are complex and are affected by multiple interacting variables whose values cannot be controlled and whose history is frequently unknown. Therefore, relationships between environment, phenotype and genotype are difficult to establish through this strategy, which causes that many basic questions remain unanswered. These include the effect of mutations on fitness, the relative contribution of natural selection and genetic drift, how diverse and reproducible are adaptive pathways, whether error rate is a character subjected to the action of natural selection, or how interactions between mutations and mutants influence adaptation. Experimental evolution arises in this context of uncertainty to provide a framework in which variables can be controlled by the experimenter, allowing a more precise relationship between environmental changes and the response of organisms to them (Bell, 2008; Elena et al., 2008; Kawecki et al., 2012; Elena, 2016; Roux et al., 2016; Geoghegan and Holmes, 2018; Van den Bergh et al., 2018). Populations used in these studies must evolve fast and be easy to handle, two conditions met by many microorganisms, including viruses.

In addition to all exposed above, viruses, and particularly those with an RNA genome, can be a good model for the study of the molecular evolution processes that took place before the emergence of cellular life, when natural selection acted on primitive RNA or RNA-like replicators. RNA viruses, together with viroids and some subgenomic elements, are the only current biological entities that use RNA to store genetic information. This RNA, although copied by protein enzymes encoded in the viral genome, is actively involved in its own replication, not only by providing binding sites for enzymes and ligands, but also because its copying capacity depends on the three-dimensional structure it adopts according to its primary sequence. Finally, it is widely documented that RNA viruses form population structures in quasispecies, similar to those described theoretically for the ensembles of primitive replicators. All this, together with their lower complexity with respect to cellular systems, means that RNA viruses can be considered a suitable model for the study of evolution in the hypothetical RNA world.

It is impossible to include in this review all the evolutionary questions that viruses have helped to understand. Here we do not intend to make an exhaustive list, and we will simply describe some illustrative examples.

### Effect of mutations on fitness

This question has been the focus of many experimental and theoretical studies (Cuevas et al., 2012; Vale et al., 2012; Acevedo et al., 2014; Bataillon and Bailey, 2014; Minicka et al., 2017). Domingo-Calap and Sanjuán (2011) carried out a comparative study of three phages with a ssRNA genome and three others with a ssDNA genome. Their findings showed that mutations usually have negative effects and confirmed that RNA viruses accumulated mutations at a faster rate than DNA viruses (Sanjuán et al., 2004; Carrasco et al., 2007; Domingo-Calap et al., 2009).

Small genomes, as such of RNA viruses, contain highly compacted information in which mutations are more prone to interact than in larger genomes. This kind of interactions, called epistasis, can make it very difficult to determine the relative contribution of particular mutations to the observed changes in fitness. Epistatic effects can change depending on the environment, as it was demonstrated in a study carried out with Tobacco Etch Virus that compared the effect of pairs of mutations in different hosts (Cervera et al., 2016). Epistasis may also determine evolutionary trajectories, depending on the first mutations that arise during adaptation (Zhao et al., 2019). A particular case of epistasis are compensatory mutations that are neutral or deleterious mutations that turn beneficial in the context of other deleterious mutations. Their existence is widely documented in drug-resistant mutants where the fitness cost that usually has the resistance mutation is compensated by the acquisition of new mutations (Buckheit, 2004; Boucher et al., 2009).

### Standing genetic diversity vs. diversity generated *de novo*

An evolutionary relevant question is the relative importance for adaptation of the mutations generated *de novo* vs. those pre-existing in populations. Quantitative genetics studies predict that, initially, natural selection acts on the pre-existing genetic variation, while mutations generated *de novo* are more relevant in the long term. The high genetic diversity contained in RNA virus populations provides an excellent system to study this question.

It has been shown that minority genomes, which had selective advantages against previous selective pressures, can speed up adaptation when the population faces a similar condition (Ruíz-Jarabo et al., 2002; Briones and Domingo, 2008). Recent studies carried out with hepatitis C virus showed that the diversification of the mutant spectrum that takes place when it is propagated under constant conditions included the presence of some variants that were resistant to particular antiviral drugs to which the virus had not been previously exposed (Gallego et al., 2020). Other study showed that populations of bacteriophage Qβ propagated at 37°C contain a considerable fraction of low-frequency mutations that may facilitate adaptation when the virus is exposed to 43°C (Somovilla et al., 2022). The conclusion is that the spread of populations on the space of sequences is unavoidable and play an essential role in adaptation (Koelle et al., 2006; Quakkelaar et al., 2007).

### Influence of error rate on adaptation

High error rates can constitute a great advantage for RNA virus adaptation. However, since most mutations are deleterious, a possible consequence is that small increases in the error rate could lead to the generation of increasingly unfit populations, which could ultimately become extinct. There are few studies on the effect of environmental conditions on the virus error rate, which would be highly relevant to determine its importance for virus permanence in a particular environment. In contrast, there are many experiments in which the error rate has been artificially modified through the use of mutagens (Domingo et al., 2021). This kind of assays has led to considerable progress in the knowledge of the relationships between error rate, adaptive capacity and the risk of population extinction. Many of the experiments performed in this context were inspired in the concept of error catastrophe of quasispecies theory (Biebricher and Eigen, 2005). It was found that, although in most cases viral populations eventually lost their infectivity when treated with mutagens, no evidence was found of the predicted loss of genetic information. Rather, what seemed to happen was a progressive decrease in fitness due to the increased accumulation of deleterious mutations (Bull et al., 2007).

In some cases, viral populations subjected to mutagenic action were able to select mutants with higher fidelity than the original virus (Pfeiffer and Kirkegaard, 2003; Agudo et al., 2010). Sometimes, these mutations only manifested their effect in the presence of the mutagen (Cabanillas et al., 2014), but in others, the increase in fidelity was maintained regardless of whether the mutagen was present or not (Agudo et al., 2016). These results are highly relevant, since they indicate that the error rate is a character subjected to the action of natural selection, and can be modified. Subsequent experiments carried out with fidelity mutants showed that they had a disadvantage in some infections (Vignuzzi et al., 2006; Coffey et al., 2011), suggesting that the presence of a complex mutant spectrum is necessary for the expression of certain phenotypic traits that require cooperation between different components of the population.

Finally, studies carried out with mutagens have made it possible to identify a new pathway to extinction based on the increase of defective interactions in the mutant spectrum (Grande-Pérez et al., 2005).

### Adaptation to adverse conditions in the extracellular medium

For a virus, as for primitive replicators, the capacity to resist adverse environmental conditions in the inter-replication periods is as important as the capacity to replicate successfully. The ability of viruses to increase their stability in the environment has been explored in several studies (McGee et al., 2014; Costello et al., 2015; Hanson et al., 2015; Lázaro et al., 2018; Whittington and Rokyta, 2019), as well as the possible trade-offs between increased stability and the virus multiplicative capacity (De Paepe and Taddei, 2006; Dessau et al., 2012). A study carried out with vesicular stomatitis virus evolved through a regime that involved an increase in the time that the virus spent out of the host showed the selection for virus variants with increased extracellular survival and lower fecundity (Elena, 2001; Wasik et al., 2015), which seemed to confirm the trade-off. In contrast to this, other studies carried out with phages − propagated through successive cycles of exposure to adverse extracellular conditions followed by replication at optimal conditions − showed that it was possible to increase the resistance to the harsh extracellular environment without apparent trade-offs on virus replication (McGee et al., 2014; Lázaro et al., 2018).

## Discussion

For the first time in human history, in recent decades we have reached a technological level that allows us to investigate the possible existence of life on other worlds. This is a huge challenge that, if achieved, would help us understand the meaning of life and which of its properties are essential and must be present in any of its manifestations, be they terrestrial or extraterrestrial. The search for life outside of Earth is inevitably biased by the properties of the life we know. Although this fact introduces limitations, it also provides a starting point that can guide us, provided that we are able to stop thinking about the concrete manifestations of life on Earth and start thinking in terms of the processes that life carries out. In this sense, life can be understood as a process that transforms energy using a genetic substrate to store the instructions on how to do it. Viruses do not transform energy by themselves, but they contain in their genome all the necessary instructions to manipulate the metabolism of the cells they infect, so that they manage to multiply and transmit between cells and organisms. Ultimately, the idea of metabolic autonomy is nothing more than an illusion, since, to a greater or lesser degree, most of the living beings on Earth depend on others to exist.

The transition between living and inert matter was probably a continuous process in which it is not clear where to draw the dividing line. In addition to viruses, on Earth there is a set of biological molecules –viroids, transposons, plasmids, prions– that are capable of moving between cells and organisms, producing copies of themselves with a certain independence, and that interact with life in multiple ways, accelerating its evolution and contributing to shaping it. If we want to understand life in its broadest sense, all these entities must be included in the field of study of astrobiology. We do not know if the existence of a biosphere without viruses or similar pathogens is possible, but if this were so, it would almost certainly be less rich and diverse than the one we know. As we have widely described in this review, viruses can help understand the origin, evolution, distribution, and future of life. Therefore, we encourage the astrobiology community and the funding agencies to give more support to the research on these fascinating entities, as well as to progress in the design of techniques that allow us to include viruses as signatures of the existence of life.

## Author contributions

Both authors designed the structure of this review, wrote the text, carried out the bibliographic search, reviewed the manuscript, and approved the submitted version.
